# Phenotype Report on Patients with Congenital Factor V Deficiency in Southern Iran: Recent Ten Years’ Experience

**DOI:** 10.4274/tjh.2016.0448

**Published:** 2017-08-02

**Authors:** Mohammad Mostafa Safarpour, Sezaneh Haghpanah, Aidin Meshksar, Mehran Karimi

**Affiliations:** 1 Shiraz University of Medical Sciences, Hematology Research Center, Shiraz, Iran

**Keywords:** Congenital, Factor V deficiency, phenotype, Rare bleeding disorders

## Abstract

This study aimed to investigate clinical symptoms in patients with congenital factor V (FV) deficiency and the relationship between phenotype and factor activity level. Thirteen patients with congenital FV deficiency were investigated and the factor activity level and first clinical presentations were studied for each patient. The most common first signs and symptoms were post-surgery, post-partum, post-circumcision, and post-traumatic bleeding (30.76%), followed by easy bruising in 23.10% of the patients. The median age at the onset of clinical signs was 18 (range: 1-53) years. Patients were categorized into two groups of major and minor bleeding based on their first clinical bleeding symptoms. There was not a significant difference between the two groups with regard to factor activity level, age at diagnosis, prothrombin time, partial thromboplastin time, and international normalized ratio (p>0.05). There is a discrepancy between plasma FV activity level and the severity of clinical presentations.

## Abstract (tr)

Bu çalışma konjenital faktör V (FV) eksikliği olan hastalarda görülen klinik semptomları ve fenotipik özellikler ile faktör aktivite düzeyleri arasındaki ilişkiyi araştırmayı amaçlamıştır. Konjenital FV eksikliği olan on üç hasta incelenmiş ve faktör aktivite düzeyi ile ilk klinik başvuru özellikleri her bir hasta için irdelenmiştir. En sık görülen ilk belirti ve bulgular cerrahi, doğum, sünnet ve travma sonrası gelişen kanama (%30,76) ve bunları takiben hastaların %23,1’inde görülen kolay morarmaydı. Klinik bulguların ortaya çıktığı ortanca yaş 18 idi (aralık: 1-53 yaş). Hastalar ilk klinik belirtilerine göre majör ve minör kanama grubu olarak ikiye ayrıldı. Her iki grup arasında faktör aktivite düzeyi, tanı yaşı, protrombin zamanı, parsiyel tromboplastin zamanı ve uluslararası normalleştirilmiş oran açısından anlamlı bir farklılık yoktu (p>0,05). Plazma FV aktivite düzeyleri ve klinik başvuru şiddeti arasında bir tutarsızlık olduğu görüldü.

## INTRODUCTION

Factor V (FV) is a 330-kDa glycoprotein that is synthesized in the liver and then released into the blood circulation. Eighty percent of the factor is found in the plasma and the remainder in platelets. Activated FV plays a role as the cofactor of activated factor X (FX) in prothrombin activation and improves the formation of thrombin [1]. The gene that encodes FV consists of 25 exons and 24 introns on 1q23 [2].

In addition to the liver, megakaryocytes can produce FV, but the origin of platelets’ FV is the plasma pool. Plasma FV enters the megakaryocytes in the bone marrow and is stored in the α-granules, and then several changes occur that make FV different from the plasma form structurally and functionally. Platelet FV activation is enhanced by activated FXa or thrombin after exposure on the platelet surface and it resists activated protein C-catalyzed inactivation [3]. Plasma levels of tissue factor inhibitor are significantly decreased in FV-deficient plasma, which causes improved thrombin generation, especially at very low FV levels (<2%) [2].

Congenital FV deficiency is one of the rare bleeding disorders with the prevalence of 1 in 1,000,000 people and an autosomal recessive inheritance pattern [1]. Consanguinity is seen among the families of the patients affected by FV deficiency [2]. FV deficiency may lead to mild or severe bleeding and presents with bruising, epistaxis, etc. as well as hemarthrosis and intracranial hemorrhage (ICH) in severe forms [4]. Heterozygous FV deficiency is difficult to diagnose because it does not cause any increase in bleeding risk or significant prolongation of coagulation tests [5].

Acquired FV deficiency is a rare condition that occurs post-operation, post-partum, and following specific autoimmune disorders and malignancies [2].

Severe bleeding manifestations are usually limited to the patients with factor level of less than 1%, but it seems that there is no clear-cut relationship between the plasma factor activity level and the phenotype. Some FV-deficient patients are completely asymptomatic for several years in spite of their undetectable plasma FV levels [6].

Fresh frozen plasma (FFP) is used as a remedy of congenital FV deficiency. Although FV concentrate was recently developed by Kedrion Company, Italy (www.kedrion.com/product-development), it is still not Food and Drug Administration-approved. It seems that both Octaplas (solvent/detergent-treated pooled plasma) and Octaplas TP (Octaplas and FP24 (plasma frozen within 24 h), melted and stored for 5 days) can improve the EXTEM clotting time results equally and are alternative treatments to FFP in FV-deficient patients [7].

Our aim in this study is to evaluate phenotype findings in patients with congenital FV deficiency and any probable relationship between the factor level in plasma and the severity of symptoms of patients in southern Iran.

## MATERIALS AND METHODS

**Patients**

A cross-sectional study was conducted at the Shiraz Hemophilia Center, Shiraz, southern Iran, from February to May 2015. Peripheral citrated blood (10-15 mL) of 13 affected individuals was collected after the diagnosis of FV deficiency in accordance with the Helsinki Declaration. Seven patients were the offspring of consanguineous marriages (patient numbers 2, 4, 5, 7, 8, 10, and 11). Cases 7 and 8 were relatives (brother and sister). The father and sister of case 10 were also affected. Informed consent was received from all individuals. The Ethics Committee of Shiraz University of Medical Sciences approved the study. A questionnaire was designed to collect all demographic data of patients including age, sex, factor activity level, type of bleeding, and treatment.

### Coagulant Activity

FV activity was measured at the clinical laboratory of the Hemostasis and Thrombosis Genetic Center using one-stage assays (HemosIL kits, Instrumentation Laboratory Company, Milan, Italy) and IL 9000 (Instrumentation Laboratory Company). FVIII activity was also measured in all patients to rule out combined FV and FVIII deficiency. Based on the bleeding types, patients were divided into two groups: major bleeding (defined as hemarthrosis, gastrointestinal bleeding, post-traumatic and post-surgery bleeding, any other bleeding events causing decreased hemoglobin level at least 2 g/dL or more from baseline, and ICH) and minor bleeding (defined as epistaxis, gingival bleeding, hematoma, post-trauma or post-surgery bleeding causing decreased hemoglobin level of less than 2 g/dL from baseline, and easy bruising). Viral hepatitis status (HCV, HIV, and HBV) and inhibitor levels of all patients were checked every 6 months and biochemistry tests including liver function tests were performed annually.

### Statistical Analysis

Data were analyzed with SPSS 21 (IBM Corp., Armonk, NY, USA). Descriptive data were presented as median and interquartile range (IQR). Quantitative data were compared between the two groups of patients by Mann-Whitney test. P<0.05 was considered statistically significant.

## RESULTS

### Phenotype Analysis

Clinical and laboratory findings of patients with congenital FV deficiency are presented in Table 1. The median age of the patients was 18 years (IQR=30 years), ranging from 1 to 53 years old, with a male-to-female ratio of 9:4. The median factor activity level was 4% (IQR=4%) and none of the patients had inhibitor abnormalities. All the patients had been on on-demand therapy with FFP (10-15 mL/kg) except one patient developing ICH who had been on prophylaxis with 15 mL/kg FFP twice a week with no major bleeding after administration. All the patients had negative viral markers and normal FVIII activity levels and liver function tests.

The most common first signs and symptoms were post-surgery, post-partum, post-circumcision, and post-traumatic bleeding (30.76%), followed by easy bruising in 23.10% of the patients. Two patients (15.38%) presented with ICH, one of whom died. Two other patients (15.38%) were cases of post-dental extraction and gingival bleeding. One (7.69%) patient was detected during pre-operation laboratory data evaluation and another one (7.69%) had epistaxis.

Patients were categorized into two groups of major (n=7) and minor (n=6) bleeding based on the first clinical bleeding symptoms. Table 2 summarizes the comparison of age and laboratory data between the two groups of patients with major and minor bleeding. There was no statistically significant difference between the two groups with regard to factor activity level, age at diagnosis, prothrombin time (PT), partial thromboplastin time (PTT), or international normalized ratio (p>0.05).

## DISCUSSION

Despite the fact that congenital FV deficiency is a rare bleeding disorder, a decent amount of information is now available about the phenotype and clinical presentations of the disease due to several studies on this issue. Our study showed wide heterogeneity in patients with congenital FV deficiency. Due to this heterogeneity, complete screening of FV deficiency phenotypes and determination of a probable relationship among phenotype, age at first clinical presentation, and plasma FV level is needed. The relationship between phenotype and plasma FV levels has not been clearly identified yet. Our study showed no significant association between the plasma factor activity level and the phenotype of our patients. The variability in phenotype is possibly due to differences in the genetic background, such as pleomorphism of mutations in the same gene [8]. The cause of this discrepancy is largely unknown; however, different investigations and studies suggest that platelets may play a significant role. Tissue factor inhibitor plasma levels are also decreased in FV-deficient patients, which causes considerably enhanced thrombin generation at very low FV levels of less than 2% [2]. On the other hand, combined FV and FVIII deficiency should be considered in the event of prolongation of both PT and PTT [9], which was ruled out here by normal FVIII levels in all affected patients.

Clinical presentation of FV deficiency has a range of mild to severe bleeding that is unrelated to factor level. Diagnosis is based on the measurement of plasma factor level and prolonged PT and activated PTT [2]. Residual intraplatelets FV contribution can also be assessed by whole-blood rotation thromboelastometry [10,11].

In accordance with the previous reports, our study showed that post-traumatic bleeding, easy bruising, and epistaxis are common symptoms among the patients who suffer from congenital FV deficiency. According to the occurrence of severe symptoms such as ICH in these patients (patient numbers 12 and 13), it seems that a prophylaxis regimen is mandatory in patients with severe symptoms or severe deficiency of factor activity level. No relationship between factor activity level and phenotype of the disease was found.

The best option for treatment of these patients is factor concentrate, which was not available at our center; as such, FFP was the only optional management. However, we did not see any viral hepatitis, confirming the safety of FFP in our region.

## CONCLUSION

Our study identified that there is no relationship between the plasma factor level and phenotypes of the patients. Therefore, the factor activity level cannot be used to predict the clinical presentation severity. This means that efforts should be focused on the early diagnosis of the disorder to perform prophylactic approaches in severe cases and administer the best methods of treatment to prevent major complications. However, our sample size was small and further studies with larger sample sizes are suggested to confirm these findings.

## Figures and Tables

**Table 1:**
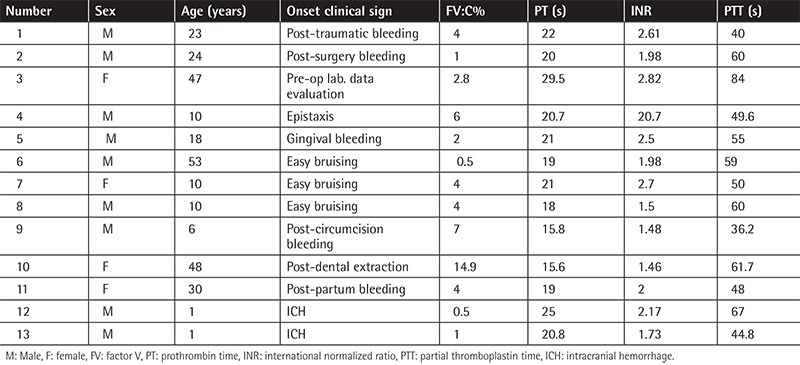
Clinical and laboratory findings of patients with congenital factor V deficiency.

**Table 2:**
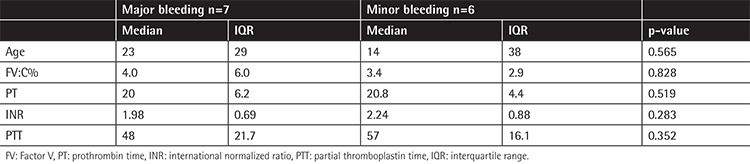
Comparison of age and laboratory data between the two groups of patients based on first clinical bleeding symptom.
